# Intermittent strong transport of the quasi-adiabatic plasma state

**DOI:** 10.1038/s41598-018-26793-8

**Published:** 2018-06-05

**Authors:** Chang-Bae Kim, Chan-Yong An, Byunghoon Min

**Affiliations:** Physics Department and Research Institute for Origin of Matter and Evolution of Galaxies, Soongsil University, Seoul, 156-743 Korea

## Abstract

The dynamics of the fluctuating electrostatic potential and the plasma density couched in the resistive-drift model at nearly adiabatic state are simulated. The linear modes are unstable if the phase difference between the potential and the density are positive. Exponential growth of the random small perturbations slows down due to the nonlinear E × B flows that work in two ways. They regulate the strength of the fluctuations by transferring the energy from the energy-producing scale to neighboring scales and reduce the cross phase at the same time. During quasi-steady relaxation sporadic appearance of very strong turbulent particle flux is observed that is characterized by the flat energy spectrum and the broad secondary peak in the mesoscale of the order of the gyro-radius. Such boost of the transport is found to be caused by presence of relatively large cross phase as the E × B flows are not effective in cancelling out the cross phase.

## Introduction

In the experiments of the magnetically confined fusion plasmas, the confinement is degraded by the presence of the turbulence. Long-range electromagnetic interactions between the charged particles cause the non-uniform plasma equilibrium linearly unstable. Small perturbations grow exponentially and the plasma becomes turbulent through numerous nonlinear interactions, notably the *E* × *B* advection. Turbulence enhances the level of the transport of the particles and the heat much higher than classical collisional dissipation from the hot-and-dense core to the edge. Since there exist various forms of the free-energy source that may be released to turbulence, it is a general practice to study the effect of each source on the transport separately. At the edge region of the confined plasma, the plasma temperature is not as high as the core so that the collisions between the plasma particles are not negligible in the plasma dynamics.

Set of Hasegawa-Wakatani equations^1^ is a minimal model that is suitable for the study of the edge plasma confined under strong magnetic field. At the equilibrium the electron temperature, which is isothermal, is much higher than the ion temperature and the particle density is non-uniform with a constant gradient length across the radius. After small random disturbance is introduced to the equilibrium, the evolutions of the fluctuating electric potential and the electron density are studied with fixed density gradient. The free energy associated with the non-uniform density is tapped into the plasma by the particle transport across the plane perpendicular to the magnetic field which is then dissipated by the resistive dissipation along the field. The plasma is assumed to be a slab where the Cartesian coordinates $x$ and *y* represent the radial and the poloidal positions, respectively. The plasma is linearly unstable if the density perturbation of the resistive-drift wave lags behind the potential perturbation. The linear growth rate is large at the scale of the order of the ion gyro-radius *ρ*_*s*_ that is associated with the electron temperature. The fact that the stability has a strong dependence on the cross phase between the perturbations has a certain similarity to a damped harmonic oscillator enforced by the external harmonic force. The power supplied to the oscillator is proportional to *sin*^2^Δ where Δ is the phase difference between the force and the displacement. The similarity between the two problems ends here because the potential and the density in the plasma are coupled so that they are determined self-consistently by the dynamics. The strength of the perturbation and the particle transport Γ, which is the product of both the particle density *n* and the *E* × *B* velocity *v*_*Ex*_, initially grow exponentially, because of the linear instability. With the increase of the fluctuation level, the nonlinear *E* × *B* advection becomes important to the evolution of the fluctuations. The energy of the fluctuation becomes saturated and the peak of the energy spectrum moves toward larger scale than *ρ*_*s*_. Since Γ depends both on the fluctuation levels of the potential and the density and on the cross phase^2–4^ between them, Γ declines at a faster rate than the energy. It is because the cross phase relaxes toward 0 as larger-scale fluctuations carry most energy.

While running simulations of the resistive-drift plasma dynamics^1^, where the fluctuating electron density closely follows the Boltzmann distribution, an interesting result that had not been reported before was discovered during the quasi-steady period of the saturation. It is found that the saturation of Γ does not proceed in monotonic fashion. Instead, the fast-pace increase and decrease of Γ is found to repeat intermittently during the relaxation stage. Complete explanation of the observed non-uniform evolution of Γ would require quantitative information on how the energy and the phase, which are of course coupled to each other, evolve under the *E* × *B* energy fluxes. As the analytical evaluation of the nonlinear fluxes is of higher degree of difficulty, we intend to rely on the numerical data to elucidate their impact on the cross phase. A brief account of the results is as follows.

**Figure 1:**
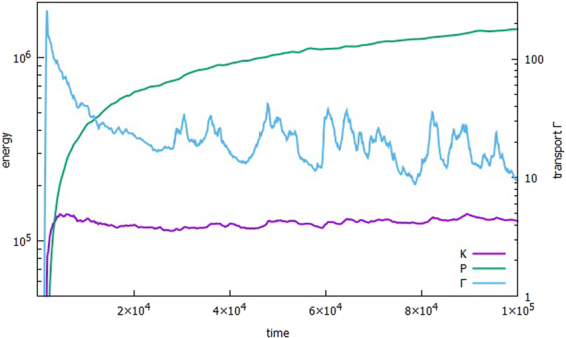
Evolutions of the kinetic energy $K=\sum _{\overrightarrow{k}}\frac{1}{2}{|k{\phi }_{\overrightarrow{k}}|}^{2}$, the particle-density energy $P=\sum _{\overrightarrow{k}}\frac{1}{2}{|{n}_{\overrightarrow{k}}|}^{2},$ and the transport up to *t* = 10^5^.

Figure 1 shows that in the early stage of the relaxation when the energies reach a plateau or change slowly, Γ monotonically decreases with shorter time scale. It may be suspected that the relaxation of the phase takes longer than the fluctuation intensity. When Γ is intermittently large at the later stage of the relaxation, its spectrum displays a distinct hump in the mesoscale, while the energy spectrum in the scale does not puff up, but is almost flat, or its gradient is less steep. This suggests that δ, which is the cross phase between the electric potential and the density, is not uniform across the spectral range, and that in particular, δ changes abruptly near the foot of the hump. Since *E* × *B* nonlinearity clearly controls the behavior of δ during the relaxation, it is natural to separate out its effect on δ. It may be better to divide δ into *δ*_0_ and *δ*_1_, where *δ*_1_ is driven by an advective part *Q* of the nonlinear *E* × *B* energy flux, and *δ*_0_ is the rest that includes the linear response. During the course of early development of the energy saturation, *δ*_0_ is almost cancelled out by *δ*_1_ in the scale where most energy is contained. As a result, Γ decreases continuously in time, until slowly changing *Q* turns the corner to make *δ*_1_ ineffective in blocking *δ*_0_ in the mesoscale. Then, a little hump in the spectra of Γ starts to grow, and it becomes larger as *δ*_1_ adds to, instead of subtracting from, *δ*_0_, as *Q* changes sign. After *Q* reaches a peak, it goes on decreasing, to change the sign. Then, the hump in the spectrum becomes lower, and drags Γ down. The same process repeats itself during the quasi-steady phase of the energy saturation.

There exist many reports of the experimental measurements of the cross phase^2,3^. In spite of its importance in the plasma transport by the drift-wave turbulence^4^ the cross phase has not been studied theoretically as much as the energetics of the fluctuations. For example, the spectrum of the fluctuations was theoretically predicted in refs^5,6^ and the energy transfer between the fluctuations was analyzed by Manz^7^. Naulin^8^ tried to compute the cross phase by modelling the non-adiabatic response. Camargo^9^ considered the cross phase in the linearly unstable regime. The role of *E* × *B* energy flux *Q* on the cross phase has been touched upon in the context of the turbulence under sheared flows. Terry^10,11^ estimated the suppressions of the turbulence and the cross phase by the flow shear. Experimental evidence that the sheared flow modifies the turbulent transport through the change of the cross phase has been reported^12,13^. Impact of the zonal flow on the cross phase was reviewed by Diamond and co-workers^14^.

The role of *Q* fluxes is not limited to the particle transport. For example, they are equally important in the thermal energy transport because it depends on the cross phase between the electric potential and the pressure. We proceed by describing the evolutions of the energy and the cross phase based on the formalism that was recently developed^15^, followed by the presentation of the scenario based on the analyses of the numerical simulation, and the summary.

## Results

### Description of the model dynamics

The dynamics of the plasma are pedagogically described by the resistive-drift model that couples the evolutions of the electrostatic fluctuations of the electric potential ϕ and the electron density *n*. Through the model one may study the particle transport while the gradient of the background electron density is held fixed with the constant scale length *L*_*n*_. Since the electron temperature does not fluctuate, the heat transport of the plasma is excluded in the model. Collisions between the ions and the electrons are included so that the steady state is achieved where the free tapped into the plasma is dissipated by the resistive loss. Utilizing the normalizations of the spatial scale *ρ*_*s*_, the time *L*_*n*_/*c*_*s*_, ϕ and *n* with (*T*_*e*_*ρ*_*s*_/*eL*_*n*_) and *n*_0_*ρ*_*s*_/*L*_*n*_, respectively, where ${c}_{s}=\sqrt{{T}_{e}/{M}_{i}}$ is the sound speed and *ρ*_*s*_ = *c*_*s*_/Ω_*i*_, we have:

1 $$[{\partial }_{t}+(\hat{z}\times {\nabla }_{\perp }\phi )\cdot {\nabla }_{\perp }]{\nabla }_{\perp }^{2}\phi =\alpha (\phi -n)-\nu {\nabla }_{\perp }^{6}\phi ,$$

2 $$[{\partial }_{t}+(\hat{z}\times {\nabla }_{\perp }\phi )\cdot {\nabla }_{\perp }]n=-\,{\partial }_{y}\phi +\alpha (\phi -n)-\nu {\nabla }_{\perp }^{4}n$$

The plasma is assumed to be a slab where the coordinate axes are chosen so that the equilibrium density gradient is in the −*x* direction, the magnetic field along the *z* axis, and the electron diamagnetic drift along the *y*. Equation (1) describes the quasi-neutrality that the ion polarization current is balanced by the parallel electron current, where the parameter α quantifies the degree of closeness to the adiabatic state. Larger α means that the electron distribution is closer to be Boltzmann distribution. Equation (2) represents the electron continuity, as the perpendicular compression on the left-hand side equals the parallel compression. Hyper dissipations with coefficient ν are introduced to truncate the fluctuations of fine scale compared to *ρ*_*s*_^16,17^.

The energy-conservation laws in the Fourier space may be found by multiplying Eqs (1) and (2) by the complex-conjugates $-{\phi }_{\overrightarrow{k}}^{\ast }$ and ${n}_{\overrightarrow{k}}^{\ast }$, respectively, and by taking the real parts, as follows:

3 $${\partial }_{t}{K}_{\overrightarrow{k}}+ {\mathcal R} ({Q}_{\overrightarrow{k}}^{\phi })=-\,2\alpha {P}_{\overrightarrow{k}}{\beta }_{\overrightarrow{k}}({\beta }_{\overrightarrow{k}}-cos{\delta }_{\overrightarrow{k}})-\,2\nu {k}^{4}{K}_{\overrightarrow{k}},$$

4 $${\partial }_{t}{P}_{\overrightarrow{k}}+ {\mathcal R} ({Q}_{\overrightarrow{k}}^{n})={{\rm{\Gamma }}}_{\overrightarrow{k}}-2\alpha {P}_{\overrightarrow{k}}(1-{\beta }_{\overrightarrow{k}}cos{\delta }_{\overrightarrow{k}})-2\nu {k}^{4}{P}_{\overrightarrow{k}}$$

where, ${K}_{\overrightarrow{k}}=\frac{1}{2}{|k{\phi }_{\overrightarrow{k}}|}^{2}$ is the kinetic-energy density, ${P}_{\overrightarrow{k}}=\frac{1}{2}{|{n}_{\overrightarrow{k}}|}^{2}$ the particle-density energy, the relative magnitude ${\beta }_{\overrightarrow{k}}=|{\phi }_{\overrightarrow{k}}|/|{n}_{\overrightarrow{k}}|$, the phase difference ${\delta }_{\overrightarrow{k}}={\theta }_{\overrightarrow{k}}^{\phi }-{\theta }_{\overrightarrow{k}}^{n}$ with ${\theta }_{\overrightarrow{k}}^{\phi }=ta{n}^{-1}[\Im ({\phi }_{\overrightarrow{k}})/ {\mathcal R} ({\phi }_{\overrightarrow{k}})]$ and ${\theta }_{\overrightarrow{k}}^{n}$ correspondingly, and the particle transport ${{\rm{\Gamma }}}_{\overrightarrow{k}}=2{k}_{y}{P}_{\overrightarrow{k}}{\beta }_{\overrightarrow{k}}\,{\sin }\,{\delta }_{\overrightarrow{k}}$. In Eqs (3) and (4), ${Q}_{\overrightarrow{k}}^{\phi }=-\,{\phi }_{\overrightarrow{k}}^{\ast }{({\overrightarrow{v}}_{E}\cdot {\nabla }_{\perp }\omega )}_{\overrightarrow{k}}$ and ${Q}_{\overrightarrow{k}}^{n}={n}_{\overrightarrow{k}}^{\ast }{({\overrightarrow{v}}_{E}\cdot {\nabla }_{\perp }n)}_{\overrightarrow{k}}$ are the E × B fluxes for the kinetic and the particle-density energies, respectively, and the vorticity ${\omega }_{\overrightarrow{k}}=-{k}^{2}{\phi }_{\overrightarrow{k}}$ was used for the derivation. It is clear from Eqs (3) and (4) that, for linearly unstable fluctuations, ${\beta }_{\overrightarrow{k}} < \,{\cos }\,{\delta }_{\overrightarrow{k}}$ and ${k}_{y}{\beta }_{\overrightarrow{k}}\,{\sin }\,{\delta }_{\overrightarrow{k}} > \alpha (1-{\beta }_{\overrightarrow{k}}\,{\cos }\,{\delta }_{\overrightarrow{k}})$, respectively. As ${\delta }_{\overrightarrow{k}}\ll 1$ and $(1-{\beta }_{\overrightarrow{k}}) > {\delta }_{\overrightarrow{k}}^{2}/2$ in the case of highly adiabatic state (α ≫ 1), linearly unstable modes should follow ${\delta }_{\overrightarrow{k}} < {k}_{y}/\alpha$.

Let ${\rm{\Delta }}{k}_{1}$ be the range of *k* where the modes are most unstable. As the fluctuations grow, the nonlinear energy fluxes develop to slow down the growth of the fluctuations by taking the energy away from the range ${\rm{\Delta }}{k}_{1}$ to the modes of larger scale ${\rm{\Delta }}{k}_{2}$. While ${\mathcal R} ({Q}_{\overrightarrow{k}}^{\phi })$ at ${\rm{\Delta }}{k}_{1}$ is approximately $2\alpha {P}_{\overrightarrow{k}}{\beta }_{\overrightarrow{k}}({\cos }\,{\delta }_{\overrightarrow{k}}-{\beta }_{\overrightarrow{k}})$, it is now negative in ${\rm{\Delta }}{k}_{2}$. In order for the modes of ${\rm{\Delta }}{k}_{2}$ to stay nearly steady, ${\beta }_{\overrightarrow{k}}$ becomes larger than $cos{\delta }_{\overrightarrow{k}}$, so that $|{\phi }_{\overrightarrow{k}}| > |{n}_{\overrightarrow{k}}|$. Then, the resistive α term in equation (4) reverses sign so as to strengthen $|{n}_{\overrightarrow{k}}|$. Since ${\mathcal R} ({Q}_{\overrightarrow{k}}^{n})$ is negligibly small for the quasi-adiabatic plasma, it is too weak to hamper the increase of $|{n}_{\overrightarrow{k}}|$. For the modes in the scale of ${\rm{\Delta }}{k}_{2}$, $|{n}_{\overrightarrow{k}}|$ soon exceeds $|{\phi }_{\overrightarrow{k}}|$ and, thus, the α term in equation (3) becomes positive to raise the kinetic energy. The excessive energy is transferred to the modes of neighboring scale ${\rm{\Delta }}{k}_{3}$, and the process repeats.

One way of obtaining the equation for ${\delta }_{\overrightarrow{k}}$ is as follows^15^: First, Eqs (1) and (2) are multiplied by the complex-conjugates $-{\phi }_{\overrightarrow{k}}^{\ast }$ and ${n}_{\overrightarrow{k}}^{\ast }$, respectively. By dividing the imaginary part of each result by ${K}_{\overrightarrow{k}}$ and ${P}_{\overrightarrow{k}}$, respectively, and by subtracting from each other, one obtains:

5 $${\partial }_{t}{\delta }_{\overrightarrow{k}}={ {\mathcal L} }_{\overrightarrow{k}}+\frac{1}{2{P}_{\overrightarrow{k}}}[-\,\frac{\Im ({Q}_{\overrightarrow{k}}^{\phi })}{{k}^{2}{{\beta }_{\overrightarrow{k}}}^{2}}+\Im ({Q}_{\overrightarrow{k}}^{n})],$$

where, the terms that are directly related to ${\delta }_{\overrightarrow{k}}$ are bundled up into ${ {\mathcal L} }_{\overrightarrow{k}}$, where:

6 $${ {\mathcal L} }_{\overrightarrow{k}}={\beta }_{\overrightarrow{k}}[{k}_{y}\,{\cos }\,{\delta }_{\overrightarrow{k}}-\alpha (1+\frac{1}{{k}^{2}{{\beta }_{\overrightarrow{k}}}^{2}}){\sin }\,{\delta }_{\overrightarrow{k}}].$$

It is convenient to separate the cross phase as ${\delta }_{\overrightarrow{k}}={\delta }_{\overrightarrow{k}0}+{\delta }_{\overrightarrow{k}1}$, where ${\delta }_{\overrightarrow{k}0}$ is defined as:

7 $${\delta }_{\overrightarrow{k}0}={ta}{{n}}^{-1}\frac{{k}_{y}}{\alpha (1+\frac{1}{{k}^{2}{{\beta }_{\overrightarrow{k}}}^{2}})}.$$

After replacing ${\delta }_{\overrightarrow{k}0}$ in equation (6) by using equation (7), ${ {\mathcal L} }_{\overrightarrow{k}}$ becomes:

8 $${ {\mathcal L} }_{\overrightarrow{k}}=-{\beta }_{\overrightarrow{k}}\sqrt{{{k}_{y}}^{2}+{\alpha }^{2}{(1+\frac{1}{{k}^{2}{{\beta }_{\overrightarrow{k}}}^{2}})}^{2}}\,{\sin }\,{\delta }_{\overrightarrow{k}1}.$$

when the electron response is close to be adiabatic, ${\beta }_{\overrightarrow{k}}$ is almost unity, and ${\partial }_{t}{\beta }_{\overrightarrow{k}}$ may be approximately negligible. Equation (5) becomes

9 $${\partial }_{t}{\delta }_{\overrightarrow{k}1}=-\,\alpha (1+\frac{1}{{k}^{2}}){\sin }\,{\delta }_{\overrightarrow{k}1}+\frac{1}{2{P}_{\overrightarrow{k}}}[-\frac{\Im ({Q}_{\overrightarrow{k}}^{\phi })}{{k}^{2}}+\Im ({Q}_{\overrightarrow{k}}^{n})]$$

The first term on the right-hand side of equation (9) has the desirable property of keeping ${\delta }_{\overrightarrow{k}1}$ close to zero, if the second term is negligible. Since $\Im ({Q}_{\overrightarrow{k}}^{n})$ is small relative to $\Im ({Q}_{\overrightarrow{k}}^{\phi })$, and ${\partial }_{t}{\delta }_{\overrightarrow{k}1}\approx 0$ at quasi-steady state, ${\delta }_{\overrightarrow{k}1}$ is positive if $\Im ({Q}_{\overrightarrow{k}}^{n})$ is negative. As a result, ${\delta }_{\overrightarrow{k}}$ is larger than ${\delta }_{\overrightarrow{k}0}$, and ${{\rm{\Gamma }}}_{\overrightarrow{k}}$ is enhanced.

### Numerical results

Figure 1 shows the evolutions of the kinetic energy $K={\sum }_{\overrightarrow{k}}{K}_{\overrightarrow{k}}$ and the particle-density energy $P={\sum }_{\overrightarrow{k}}{P}_{\overrightarrow{k}}$ up to *t* = 10^5^. The exponential growths of *K* and *P* due to the linear instability in the range *kρ*_*s*_≤1 begin to slow down around *t* = 1.7 × 10^3^. *K* becomes steady at *t* ≈ 5 × 10^3^, when the *E* × *B* energy flux ${\mathcal R} ({Q}_{\overrightarrow{k}}^{\phi })$ counters the destabilizing α term in equation (1), to move the spectral peak of *K* to *kρ*_*s*_ ≈ 0.1. On the other hand, *P* is still increasing beyond *t* = 10^5^, albeit slowly, because ${\mathcal R} ({Q}_{\overrightarrow{k}}^{n})$ is so weak for highly adiabatic state (α = 10) that the relocation of ${P}_{\overrightarrow{k}}$ to reach saturation takes longer^18^. Also plotted in Fig. 1 is the particle transport ${\rm{\Gamma }}={\sum }_{\overrightarrow{k}}{{\rm{\Gamma }}}_{\overrightarrow{k}}$ on the right vertical axis. Unlike *K* and *P*, there seem to be two stages in the evolution of Γ after the linear growth. First, when the energies enter the saturation phase, Γ starts to drop fast, and monotonic decay of Γ follows, as a result of both the energy peak moving toward small *kρ*_*s*_, and ${\delta }_{\overrightarrow{k}}$ gradually becoming small, due to the action of $\Im ({Q}_{\overrightarrow{k}}^{\phi })$ in equation (9). After *t* ≈ 2.5 × 10^4^, Γ stops falling, and begins the second phase of aperiodic repeat of going up and down. Around *t* = 8 × 10^4^, it jumps four times higher in time Δ*t* = 3.5 × 10^3^.

**Figure 2:**
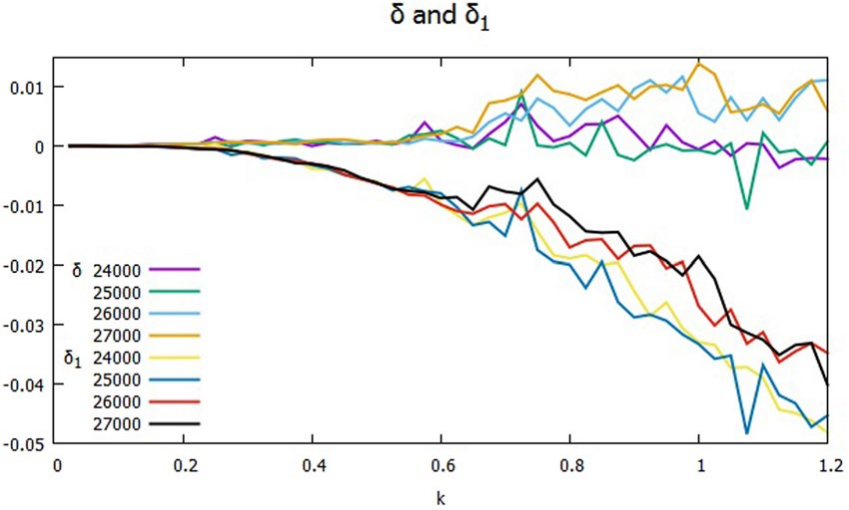
Cross phases δ_*k*_ and δ_*k*1_ during the period of the transition from the monotonic decline of Γ to the up-and-down stage, between *t* = 2.4 × 10^4^ and *t* = 2.7 × 10^4^: δ_*k*_’s in the upper group of lines, and δ_*k*1_’s in the lower.

**Figure 3:**
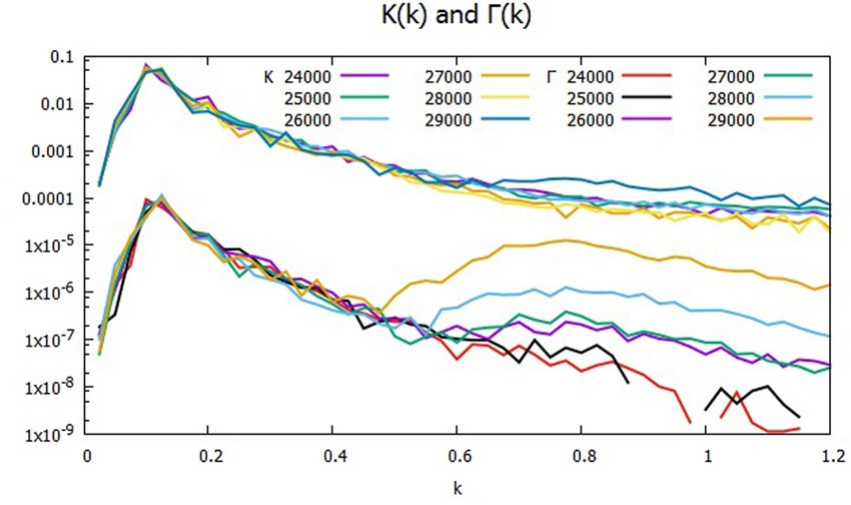
Spectra of *K*_*k*_ and Γ_*k*_ during the period of the transition from the monotonic decline of Γ to the up-and-down stage, between *t* = 2.4 × 10^4^ and *t* = 2.9 × 10^4^: *K*_*k*_’s in the upper group of plots and Γ_*k*_’s in the lower.

Figure 2 shows the cross phases at times around the transition from the monotonic decline of Γ to the up-and-down period starting at *t* = 2.4×10^4^ and ending at *t* = 2.7 × 10^4^ with the time interval *t* = 1×10^3^ over the range of *k* between 0 and 1.2. The plotted data *δ*_*J*_ and *δ*_*J*1_ are the average values of ${\delta }_{\overrightarrow{k}}$ and ${\delta }_{\overrightarrow{k}1}$, respectively, in the bin of *J* ≤ |$\overrightarrow{k}|$| < *J* + Δ*k* with the width Δ*k* = 2π/*L*. *δ*_*J*_’s are small but positively finite, while *δ*_*J*1_’s are negative, and larger than their respective *δ*_*J*_’s. Note that in the spectral range *kρ*_*s*_ ≥ 0.6, ${\delta }_{\overrightarrow{k}}$ is approximately zero at *t* = 2.4 × 10^4^, but is small but finite of order 10^−2^ at *t* = 2.7 × 10^4^. It turns out that $\Im ({Q}_{\overrightarrow{k}}^{\phi })$ has ${\delta }_{\overrightarrow{k}1}$ almost cancel out ${\delta }_{\overrightarrow{k}0}$, which is nearly constant in time, at *t* = 2.4 × 10^4^, but that it is too small to complete the cancelation at *t* = 2.7 × 10^4^. Figure 3 shows the spectra *K*_*k*_ and Γ_*k*_ of the kinetic energy and the particle transport over the half plane *k*_*y*_ ≥ 0 at the transition from *t* = 2.4 × 10^4^ until *t* = 2.9 × 10^4^. The *K*_*k*_’s are the larger of the two, and are plotted in the upper group of lines, while the Γ_*k*_’s are in the lower. Around *kρ*_*s*_ = 0.7, Γ_*k*_ is rising after *t* = 2.5 × 10^4^, and the hump becomes higher and wider onward. At *kρ*_*s*_ = 0.8, Γ_*k*_ jumps about three orders of magnitude relative to the time *t* = 2.4 × 10^4^. Meanwhile, *K*_*k*_ does not show appreciable change in time until *t* = 2.8 × 10^4^, and it becomes nearly flat at *t* = 2.9 × 10^4^ in the range. Since Γ_*k*_ drives K_*k*_ through P_*k*_ as in Eqs (3) and (4), K_*k*_ lags Γ_*k*_ in response to the change of δ_*k*_.

**Figure 4:**
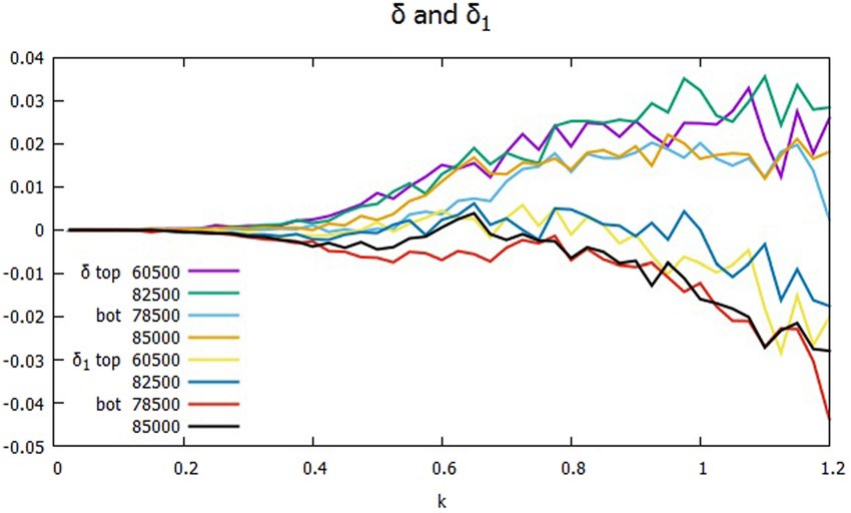
Cross phases δ_*k*_ and δ_*k*1_ at the times of strong transport (top), *t* = 6.05 × 10^4^ and 8.25 × 10^4^, and at the times of weak transport (bot), *t* = 7.85 × 10^4^ and 8.5 × 10^4^: δ_*k*_’s in the upper group of plots and δ_*k*1_’s in the lower.

**Figure 5:**
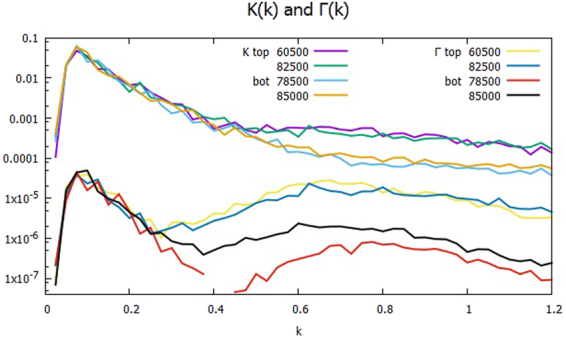
The spectra of *K*_*k*_ and Γ_*k*_ at the times of strong transport denoted as top and weak transport denoted as bot at the same time set of Fig. 4: *K*_*k*_’s in the upper group of lines and Γ_*k*_’s in the lower.

Figures 4 and 5 show corresponding plots of the cross phase δ_*k*_ and δ_*k*1_ and the spectra *K*_*k*_ and Γ_*k*_ in the up-and-down period of Γ at four different times, two each when Γ is high and low, denoted as top and bot, respectively. In Fig. 4, the differential of δ_*k*_ between the times when Γ is strong and weak is typically about 0.01. For example, at *kρ*_*s*_ = 0.8, δ_*k*_ changes from 0.01 to 0.02. In Fig. 5, the peaks of *K*_*k*_ and Γ_*k*_ are seen to move toward slightly smaller *kρ*_*s*_, and their slopes in the range 0.2 ≤ *kρ*_*s*_ ≤ 0.45 are not as steep as the transient cases of Fig. 3. The ratio of *K*_*k*_’s at *t* = 8.25 × 10^4^ (strong transport) and at *t* = 8.5 × 10^4^ (weak transport) is about five in the mesoscale. Yet the ratio of Γ_*k*_’s at the same times is of the order of 10. Therefore, it agrees with the difference of δ_*k*_ between the times. Note that at *t* = 6.05 × 10^4^ and 8.25 × 10^4^, when the transport is strong, Γ_*k*_ of the broad secondary peak is well within an order of magnitude of the primary peak around *kρ*_*s*_ = 0.1. Considering the width of the peaks and the number of modes involved, the secondary peak contributes more than the primary peak to push up the transport at *t* = (6.05 × 10^4^ and 8.25 × 10^4^). By contrast, the primary peak of *K* is still two orders of magnitude higher. As a result, Γ fluctuates more than *K* in time.

**Figure 6:**
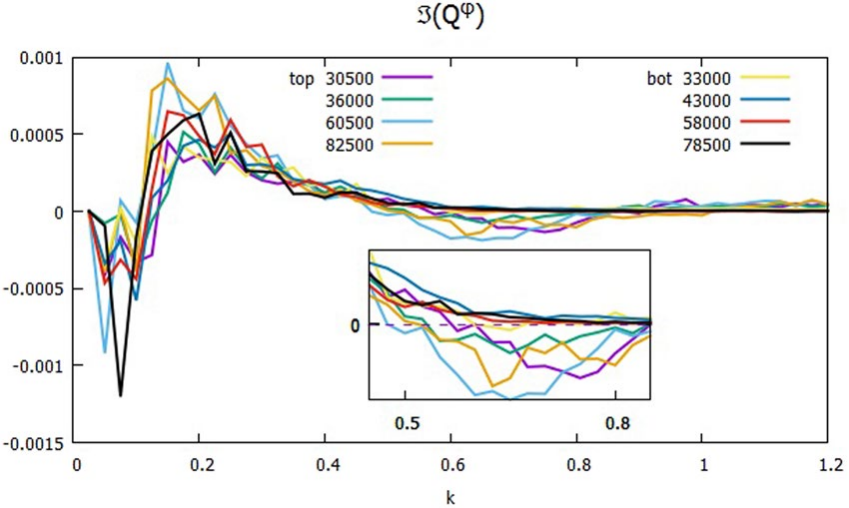
$\Im ({Q}_{k}^{\phi })$ when the transport is strong (top) and weak (bot) at the same time set of Fig. 4: Inset magnifies the plot between 0.45 and 0.85.

As noted in Fig. 4, δ_*k*1_ in the mesoscale is of opposite sign between strong and weak transport: positive in the former, and negative in the latter. As equation (9) suggests, this is because $\Im ({Q}_{J}^{\phi })$, which is the sum of $\Im ({Q}_{\overrightarrow{k}}^{\phi })$ in the bin *J* ≤ |$\overrightarrow{k}$| < *J* + Δ*k* on the half plane *k*_*y*_ ≥ 0, changes sign. Figure 6 clearly shows that in the range of *k* between 0.45 and 0.85, $\Im ({Q}_{\overrightarrow{k}}^{\phi })\ll {10}^{-4}$ is positive for low transport, whereas $\Im ({Q}_{\overrightarrow{k}}^{\phi })$ is of the order of 10^−4^, and negative for high transport. On dimensional grounds, $\Im ({Q}_{\overrightarrow{k}}^{\phi })$ may be approximated as *k*_*y*_ times a certain velocity *V*_*y*_ and *k*^2^|*φ*_*k*_|^2^, where *V*_*y*_ is interpreted as an advecting velocity^19^. *V*_*y*_ is to be negative, i.e. along the direction of the ion diamagnetic drift, in the mesoscale to achieve large transport, which Fig. 6 confirms.

## Summary

Aperiodic manifestation of very strong particle transport of the resistive-drift plasma turbulence at highly adiabatic state is understood by the coupled dynamics between the fluctuation energies and the cross phase. The *E* × *B* energy flux plays dual roles in the plasma transport. Its foremost role, which has been well studied, is the transfer and reallocation of the kinetic energy through cascade. At the same time, it can indirectly influence the evolution of the energy, by controlling the cross phase through advection. It turns out that the latter effect is strong in the mesoscale of *kρ*_*s*_, roughly between (0.5 and 0.9). As a result, the cross phase is large in the mesoscale. A secondary broad peak in the mesoscale appears in the spectrum of the plasma particle transport that exceeds the contribution of the primary peak, where most of the energy resides. The energy spectrum becomes flat, instead of decreasing, in the mesoscale, because the particle transport pushes up the plasma energy.

Although the cross phase between the fluctuations is an integral part in the determination of the transport flux, there exist not many works that deal with it on the same footing as the fluctuation energy^20^. It is stressed in this work that studying the cross phase between the electrostatic potential and the plasma density, in addition to the energy evolution, is crucial to understanding the particle transport near the adiabatic state in the Hasegawa-Wakatani resistive-drift plasma model. Since the electrons in the fusion plasmas are almost adiabatic, the particle transport may be presumed to be small. Yet, it may intermittently become strong, as indicated in the simulation. Advection of the kinetic energy in the same direction as the ion diamagnetic drift produces large cross phase and the particle transport. In other models of the plasma turbulence, if the transport and the energy relax by different rates from each other in the nonlinear saturation stage, it may indicate that the relaxation of the cross phase between the fluctuations is important in the prediction of the plasma confinement. Analytical work is desirable to supplement the present results, by approximating the advective *E* × *B* flux in terms of the energy and the cross phase, to close the dynamic chain between the energy and the cross phase. The evolution of the cross phase is believed to be similarly worked out inside the fusion plasmas. When the zonal flow is present with the turbulence, the advective *E* × *B* flux by the non-zonal fluctuations may be negligibly small compared to the advection by the zonal flow. As far as the cross phase is concerned, the latter advection is canceled out and the non-zonal *E* × *B* advection may lead to the disparity between the distributions of the fluctuations and the transport. Extensions to the transport of both the particles and the thermal energy in the plasmas with the presence of the zonal flows are in progress.

## Methods

### Numerical analyses

Eqs (1) and (2) are numerically integrated on the square domain of the length *L* = 80π that is evenly divided by *N*^2^ grid points, with *N* = 256. BOUT +  + platform^21^ is used, employing PVODE with adaptive time stepping to advance in time, and the Arakawa scheme^22^ for the treatment of the Poisson brackets. For the present work, the periodic boundary conditions are imposed, and the coefficient of the hyper-dissipation is *ν* = 3 × 10^−3^, while the adiabaticity parameter α = 10. Initially, the fluctuations are set as *n* = 0 and ${{\nabla }_{\perp }}^{2}\phi$ to be the modulation of ${10}^{-2}\,\sin (\frac{8\pi x}{L}+{\theta }_{x})\sin (\frac{8\pi y}{L}+{\theta }_{y})$ with pseudo-random phases *θ*_*x*_ and *θ*_*y*_ by 13 other small-amplitude harmonics of box size *L* in both *x* and *y*.

### Data availability

The datasets generated during the current study are available from the corresponding author on reasonable request.
